# Older and Younger Adults Reappraise Negative Life Events in Different Ways

**DOI:** 10.1093/geroni/igaa057.3294

**Published:** 2020-12-16

**Authors:** Lucas Hamilton, Eric Allard

**Affiliations:** Cleveland State University, Cleveland, Ohio, United States

## Abstract

Past reappraisal studies have been equivocal regarding age and reappraisal efficacy potentially due to the use of laboratory-generated stimuli. We examined reappraisal in a more self-relevant context: negative autobiographical events. 49 younger adults (YA) and 47 older adults (OA) generated 50 negative memories and provided negativity, positivity, and vividness ratings. One to two weeks later, participants underwent the reappraisal task during which physiological data were collected. Participants implemented one of three instructions for 30 seconds: remember naturally, increase negative reactions, or decrease negative reactions via a “positivizing” tactic. Each instruction was provided for 10 unique memories with negativity, positivity, and vividness ratings collected after each trial. 2 (Age; YA, OA) × 3 (Instruction; Remember, Increase, Decrease) mixed ANOVAs uncovered no differences in negativity or vividness ratings before reappraisal. However, OAs rated all memories more positively than YAs. This age difference persisted after reappraisal; however, OAs rated all memories more negatively and vividly than YAs, although both decreased compared to pre-reappraisal levels. Cardiorespiratory data were tested via 2 × 3 mixed ANOVAs, uncovering only a main effect of age on average heart rate. A multilevel model revealed significant variability in the time-course of pupillary responses. 2 × 3 mixed ANOVAs illustrated that reappraisal brought about faster and more frequent spikes in pupil diameter, particularly for OAs. We conclude that OAs and YAs may achieve reappraisal in different ways. Contrary to strict hedonic orientations, OAs simultaneously maintain higher negativity and positivity than YAs challenging existing propositions regarding age-related prioritization of hedonic goals.

